# Peripheral regulatory cells immunophenotyping in Primary Sjögren's Syndrome: a cross-sectional study

**DOI:** 10.1186/ar4245

**Published:** 2013-06-21

**Authors:** Janette Furuzawa-Carballeda, Gabriela Hernández-Molina, Guadalupe Lima, Yahaira Rivera-Vicencio, Karen Férez-Blando, Luis Llorente

**Affiliations:** 1 Department of Immunology and Rheumatology, Instituto Nacional de Ciencias Médicas y Nutrición Salvador Zubirán, Vasco de Quiroga No. 15, Col. Sección XVI, CP 14000, Mexico City, Mexico

## Abstract

**Introduction:**

IL-10--producing B cells, Foxp3-expressing T cells (Tregs) and the IDO-expressing dendritic cells (pDC) are able to modulate inflammatory processes, to induce immunological tolerance and, in turn, to inhibit the pathogenesis of autoimmune disease.

The aim of the study was to characterize and to enumerate peripheral IL-10--producing B cells, Tregs and pDCregs in primary Sjögren's Syndrome (pSS) patients in regard of their clinical and serologic activity.

**Methods:**

Fifty pSS patients and 25 healthy individuals were included in the study. CD19^+^--expressing peripheral B lymphocytes were purified by positive selection. CD19^+^/CD24^hi^/CD38^hi^/IL-10--producing B cells, CD4^+^/CD25^hi^/Foxp3^+ ^and CD8^+^/CD28^-^/Foxp3^+ ^Tregs, as well as CCR6^+^/CD123^+^/IDO^+ ^DCs, were quantitated by flow cytometry.

**Results:**

Immature/transitional circulating IgA^+ ^IL-10--producing B cells had higher levels in pSS patients versus control group, whereas CD19^+^/CD38^hi^/IgG^+^/IL-10^+ ^cells had lower percentage versus control. Indeed CD19^+^/CD24^hi^/CD38^hi^/CD5^+^/IL-10^+^, CD19^+^/CD24^hi^/CD38^hi^/CD10^+^/IL-10^+^, CD19^+^/CD24^hi^/CD38^hi^/CD20^+^/IL-10^+^, CD19^+^/CD24^hi^/CD38^hi^/CD27^-^/IL-10^+^, and CD19^+^/CD24^hi^/CD38^hi^/CXCR7^+^/IL-10^+ ^cells had higher frequency in clinical inactive pSS patients when compared with control group. Remarkably, only percentages of CD19^+^/CD24^hi^/CD38^hi^/CD10^+^/IL-10^+ ^and CD19^+^/CD24^hi^/CD38^hi^/CD27^-^/IL-10^+ ^subsets were increased in pSS serologic inactive versus control group (*P *< 0.05). The percentage of IDO-expressing pDC cells was higher in pSS patients regardless of their clinical or serologic activity. There were no statistically significant differences in the percentage of CD4^+^/CD25^hi^/Foxp3^+ ^Tregs between patient groups versus controls. Nonetheless, a decrease in the frequency of CD8^+^/CD28^-^/Foxp3^+ ^Tregs was found in inactive pSS patients versus controls (*P *< 0.05).

**Conclusions:**

The findings of this exploratory study show that clinical inactive pSS patients have an increased frequency of IL-10--producing B cells and IDO-expressing pDC cells.

## Introduction

Progress in clarifying cellular, molecular and biochemical processes that regulate immune response provides increasingly acceptable enlightenment for the normal status of tolerance to self-antigens that guards most humans from Ehrlich's imagined horror, autotoxicus [1]. Emerging data on the IL-10-producing B-cell subset provide fertile ground for resolving some perplexing immunological paradoxes. The immunoregulatory role of B cells in autoimmune disease was initially characterized in B cell-deficient mice immunized with a myelin basic protein peptide in complete Freund's adjuvant, where mice develop exacerbated encephalomyelitis compared to controls [2]. This Breg subset differentiates in a chronic inflammatory environment, expresses high levels of CD1d, produces IL-10, and suppresses the progression of intestinal inflammation by directly downregulating inflammatory cascades associated with IL-1β and signal transducer and activator of transcription 3 (STAT3) activation [3,4]. Lately, it has been described as a CD19^+^CD24^hi^CD38^hi ^immature/transitional B-cell subset that suppresses the differentiation of T helper (Th)1 cells in an IL-10-dependent, but TGF-β-independent manner, which requires CD80/CD86 interactions with target CD4^+ ^T cells. Remarkably, it has been shown that in patients with systemic lupus erythematosus (SLE), the CD19^+^CD24^hi^CD38^hi ^B subset produces less IL-10 in response to CD40 stimulation and is unable to inhibit Th responses, suggesting that altered cellular function of the subpopulation in SLE may impact the immune effector responses in this autoimmune disease [4]. Furthermore, in renal transplant patients, increased frequency of CD19^+^CD24^hi^CD38^hi ^has been associated with operational tolerance [5-8]. In addition, these IL-10-producing B cells favor the differentiation and maintenance of regulatory Foxp3-expressing T cells (Tregs) and may control organ-specific inflammation [3,4,9].

On the other hand, the catabolism of tryptophan, by the enzyme indoleamine 2,3-dioxygenase (IDO) expressed in plasmacytoid dendritic cells (pDCs), generates kynurenines, 3-hydroxyanthranilic, and quinolic acids, molecules with the ability to induce Th1 over Th2-cell apoptosis and to exert cytotoxic action on T, B and natural killer (NK) cells, but not on dendritic cells (DCs) themselves [10,11]. IDO has a selective sensitivity for Th1 over Th2 cells to tryptophan metabolites, suggesting a potential role for Th2 differentiation [12]. Furthermore, deprivation of tryptophan by IDO halts the proliferation of T cells at mid-G_1 _phase, which in concert with the pro-apoptotic activity of kynurenine, leads to diminishing T cell-mediated immune responses and the subsequent development of immune tolerance [13-17]. As IL-10-producing B cells, IDO-competent DCs have been shown to induce IL-10-producing Treg cells (Tr1) and CD4^+^/CD25^hi^/Foxp3^+ ^Tregs *in vivo*, and Treg-expressed glucocorticoid-induced TNF receptor (GITR), which in turn, can use IDO^+ ^DCs to expand their own population in a positive feedback loop [18-20].

Thus, quantitative and functional modifications of IL-10-producing B cells, Tregs and IDO-producing cells, might play a role in the pathogenesis and disease activity of autoimmune systemic disorders, including primary Sjögren's syndrome (pSS) [21], an autoimmune exocrinopathy characterized by chronic lymphocytic inflammation of the lacrimal and salivary glands resulting in keratoconjunctivitis sicca and xerostomia. Indeed, there are several features of systemic disease that may also involve additional organ systems. Although the glandular destruction has been shown to be mainly mediated by CD45RO^+^/CD4^+ ^T lymphocytes, chronic B cell activation and proliferation seem to play an intimate role [22]. In this vein, evidence of B cell hyperactivity, including circulating immune complexes, hypergammaglobulinemia, and/or autoantibodies, are frequently found in patients with pSS [22-25]. Moreover, it has been demonstrated that patients with pSS exhibit disturbed B-cell subset distribution in their blood [2].

In order to delineate the peripheral cell types with regulatory properties in patients with pSS, the aim of this study was to characterize and to enumerate IL-10-producing B cell subpopulations according to previous immunophenotyping studies [4,26,27], Foxp3-expressing CD4^+^/CD25^hi ^and CD8^+^/CD28^- ^T cells, and IDO-producing CCR6^+^/CD123^+ ^DCs by flow cytometry.

## Material and methods

### Patients

For this exploratory, observational, cross-sectional study, we included 50 consecutive pSS patients regularly attending the Department of Immunology and Rheumatology at the Instituto Nacional de Ciencias Médicas y Nutrición Salvador Zubirán, a tertiary care center. Eligible patients had to fulfill the American-European Consensus Group criteria for pSS [28], not meet classification criteria for any other autoimmune disease and not have concomitant lymphoma. All patients had a face-to-face interview with one rheumatologist (GH) using a standardized form, which included questions about demographic data, use of medications and symptoms. Patients may or not have been receiving prednisone or immunosuppressors at the time of the assessment; however they were not included if concomitant infection was present. Furthermore, disease activity status was assessed using the European League Against Rheumatism (EULAR) Sjögren's syndrome disease activity index (ESSDAI) [29] and Sjögren's syndrome disease activity index (SSDAI) validated scales [30]. Both indexes evaluate glandular (enlarged parotid glands) and extraglandular features such as fatigue, non-erosive arthritis, skin vasculitis, interstitial lung disease, renal involvement, neurological involvement, myositis, lymphadenopathy/splenomegaly, hematological involvement and fever. In addition the ESSDAI has a biological domain that evaluates hypocomplementemia, cryoglobulinemia, hypergammaglobulinemia or high immunoglobulin G (IgG) levels. For the present study, the patients were allocated to two groups, those with clinically active disease (*n *= 17) and those with clinically inactive disease (*n *= 33). We defined clinically active disease as the presence of one or more glandular and extraglandular features recognized by the ESSDAI and SSDAI with the exception of fatigue. If any manifestation could be secondary to other concomitant condition besides SS, the feature was not considered as part of the activity assessment. Finally patients' clinical records were carefully reviewed to collect information about the disease onset and follow-up. In addition 25 age-matched healthy donors (HD) were included as controls. HD were also interviewed to discard any known autoimmune disease, use of immunosuppressors and prednisone and concurrent infections.

The protocol was approved by the Committee of Medical Ethics (Reference number 348) and performed in accordance with the revised Declaration of Helsinki. All patients gave written informed consent to participate.

### Serologic markers

Levels of serum IgG, IgM, IgA, C3 and C4 complement proteins were determined by immunoturbidimetry, serum hyperviscosity was assessed with an Ostwald viscometer, and erythrocyte sedimentation rate (ESR) was determined by the Westergren method at the assessment. Serologic activity was defined as the presence of one or more of the following features: serum viscosity >2 AU, elevated IgG (>1,741 mg/dL), IgM (>281 mg/dL), IgA (>433 mg/dL) or decreased levels of complement C3 (<90 mg/dL) and C4 (<10 mg/dL).

### Isolation of peripheral blood mononuclear cells (PBMC)

A sample of venous blood (120 mL) was obtained from each subject. PBMC were isolated by gradient centrifugation on the Lymphoprep (Axis-Shield PoC AS, Oslo, Norway).

### B-cell purification and cytometric analysis

CD19-mAb-coated microbeads (Miltenyi Biotec, Bergisch Gladbach; Germany) were used to purify blood B cells by positive selection following the manufacturer's instructions. Purity was assessed by fluorescence-activated cell sorting (FACS) staining for the B- and T-cell markers. Thus, anti-human CD19-PE and anti-human CD3-FITC monoclonal antibodies were used. This procedure normally yielded B-cell preparations which were >95% CD19+.

### Flow cytometry

CD19^+ ^cells were surface stained with several combinations of anti-human fluorochrome-conjugated antibodies for four-color analysis. CD19^+ ^cells were stained with 5 μL of anti-CD38-PECy5-labeled (clone: HIT2), anti-CD38-PE-conjugated (clone: HIT2), anti-CD24-FITC-labeled (clone: ML5), anti-IgG-PECy5-conjugated (clone: G18-145), anti-IgM-APC-labeled (clone: G20-127), anti-CD5-APC-conjugated (clone: L17F12), anti-CD10-APC-labeled (clone: HI10a), anti-CD20-APC-conjugated (clone: 2H7), anti-CD27-APC-labeled (clone: M-T271), anti-CXRC4-APC-conjugated (clone: 12G5) (BD Biosciences, San Jose, CA, USA), anti-CXCR7 (polyclonal, Abcam Inc, Cambridge, MA, USA), anti-rabbit IgG-Cy5-labeled polyclonal antibody (Abcam Inc,) and anti-IgA-PE-conjugated (clone: 11-44-2, eBioscience, San Diego, CA, USA). CD19^+ ^cells were permeabilized with 200 μL of cytofix/cytoperm solution (BD Biosciences) at 4°C for 20 minutes, then they were stained for intracellular IL-10 with PE-conjugated-anti-IL-10 (clone: JES3-19F1, BD Biosciences) or fluorescein isothiocyanate (FITC)-labeled-anti-IL-10 (clone: BT10, eBioscience). Finally, CD19^+ ^subsets were analyzed by flow cytometry with a FACScalibur (BD Biosciences). An electronic gate was made for CD38^hi^, IgA^+^, IgG^+^, or IgM^+ ^and IL-10^+ ^or for CD19^+^/CD38^hi^/CD24^hi^, CD5^+^, CD10^+^, CD20^+^, CD27^+^, CXCR4^+ ^or CXCR7^+ ^and IL-10^+ ^cells and a total of 50,000 to 100,000 events were recorded for each sample and analyzed with the CellQuestPro software (BD Biosciences). Results are expressed as the relative percentage of IL-10-expressing B cells in each gate. As isotype controls, IgG_1_-FITC/IgG_1_-PE/CD45-PeCy5 mouse IgG_1_, k (BD Tritest™, BD Biosciences) and PE-conjugated-anti rat-IL-10 IgG (clone: R35-95, BD Biosciences) were used to set the threshold and gates in the cytometer. We ran an unstained (autofluorescence control) and permeabilized PBMC sample. Autofluorescence control was compared to single-stained cell-positive controls to confirm that the stained cells were on scale for each parameter. CaliBRITE™ 3 beads (BD Biosciences) were used to adjust instrument settings, set fluorescence compensation, and check instrument sensitivity.

To determine IDO cell expression, non-B cells (unlabeled cells obtained from the column while the magnetically labeled CD19^+ ^B cells were retained on the column) were conjugated with an anti-human CCR6-PE (clone: 11A9) and CD123-PECy5 (clone: 9F5) monoclonal antibodies (BD Biosciences). Cells were permeabilized and stained with a polyclonal sheep anti-human-IDO (Pierce Biotechnology, Rockford, IL, USA) and then with FITC-conjugated-rabbit polyclonal anti-sheep antibody (Chemicon, Temecula, CA, USA). The cell subset was analyzed by flow cytometry. As the control for FITC-labeled rabbit anti-sheep specificity staining, cells were incubated with surface antibodies and FITC-conjugated rabbit anti-sheep in the absence of sheep anti-human IDO antibody. An electronic gate was made for each of the surface markers employed. Results are expressed as the relative percentage of IDO-expressing cells in each gate.

For Tregs, non-CD19^+ ^B cells were labeled with an anti-human CD4-FITC (clone: RPA-T4) and CD25-PECy5 (clone: M-A251) or CD8α-FITC (clone: HIT-8a) and CD28-PECy5 (clone: CD28.2) (BD Biosciences). Intracellular staining was performed with an anti-human Foxp3-PE-conjugated monoclonal antibody (clone: 259D/C7) (BD Biosciences). An electronic gate was made for CD4^+^/CD25^hi ^cells or CD8^+^/CD28^-^. Results are expressed as the relative percentage of Foxp3-expressing cells in each gate. The relative percentage of IL-10-producing B cells, pDC IDO^+ ^and Foxp3^+ ^Treg circulating cells was obtained from the percentage of CD19^+^/CD38^hi^/CD24^hi^/IL-10^+^, CCR6^+^/CD123^hi ^, CD4^+^/CD25^hi ^or CD8^+^/CD28^- ^cells, respectively.

### Statistics

Descriptive statistics were performed and categorical variables were compared using the *X*² test or Fisher's exact test; for analysis of continuous variables the Student *t*-test was employed. One-way analysis of variance on ranks using the Dunn method was performed for all pairwise multiple comparison procedures. Statistical analysis was done using the SigmaStat11.2 program (Aspire Software International, Leesburg, VA, USA). Data were expressed as the median and range, or mean ± SD/standard error of the mean (SEM). *P-*values smaller than or equal to 0.05 were considered significant.

## Results

### Demographic, clinical and laboratory data

**Figure 1:**
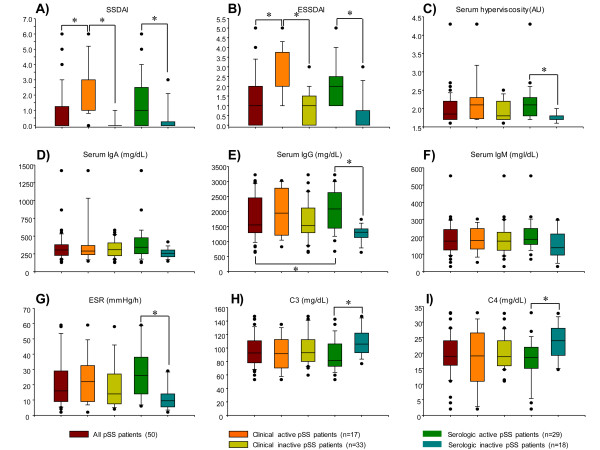
**Clinical and laboratory data for patients with primary Sjögren's syndrome (pSS)**. Results are expressed as median, 10^th^, 25^th^, 75^th^, and 90^th ^percentiles. *P < 0.05. SSDAI, Sjögren's syndrome disease activity index; ESSSDAI, European League Against Rheumatism Sjögren's syndrome disease activity index; Ig, immunobglobulin; ESR, erythrocyte sedimentation rate.

Clinical, demographic and serological variables of patients are summarized in Table 1 and Figure 1. Of 50 patients with pSS 96% percent were female with a mean age of 53 ± 12 years, median disease duration of 9.7 years (0.97 to 42.0 years), and median disease activity score of 0 (0--6) assessed by the SSDAI and 1 (0 to 5) assessed by the ESSDAI. For therapy, 22% were receiving prednisone and 38% were receiving at least one immunosuppressor as follows: antimalarial drugs, 11 patients (22%); azathioprine, 6 patients (12%); methotrexate, 5 patients (10%); and cyclophosphamide, 2 patients (4%). Seventeen patients (34%) had clinical activity manifested as parotid enlargement, vasculitis, arthritis, leukopenia, lymphopenia, pneumonitis and optic neuritis.

**Table 1:** Demographic and clinical characteristics of patients with primary Sjögren´s syndrome

Variable	Control (*n *= 25)	All pSS patients (*n *= 50)	Clinically active pSS patients (*n *= 17)	Clinically inactive pSS patients (*n *= 33)	Serologically active pSS patients (*n *= 29)	Serologically inactive pSS patients (*n *= 18)	Clinically and serologically active pSS patients (*n *= 12)	Clinically active and serologically inactive pSS patients (*n *= 4)	Clinically inactive and serologically active pSS patients (*n *= 17)	Clinically and serologically inactive pSS patients (*n *= 14)
**Demographic**										
Age (years) Mean ± SD Median Range	49.1 ± 11.0 48.5 23 to 75	53.1 ± 12.2 53.0 30 to 77	50.4 ± 11.8 48.5 30 to 73	54.4 ± 12.4 53.0 33 to 77	51.1 ± 10.0 50.0 33 to 73	55.1 ± 14.6 56.0 30 to 77	50.6 ± 10.7 48.0 33 to 73	51.5 ± 17.3 52.0 30 to 72	51.8 ± 10.3 51.5 33 to 72	56.2 ± 14.3 58.0 34 to 77
Sex (female/male)	20/5	48/2	16/1	32/1	28/1	17/1	12/0	3/1	16/1	14/0
**Clinical**										
SSDAI Mean ± SD Median Range		0.9 ± 1.5 0.0 0 to 6	2.5 ± 1.6 3.0 0 to 6	0.1 ± 0.3 0.0^a^ 0 to 1	1.3 ± 1.7 1.0 0 to 6	0.4 ± 0.85 0.0 0 to 3	2.8 ± 1.6 3.0 1 to 6	1.5 ± 1.3 1.5 0 to 3	0.2 ± 0.4 0.0 0 to 1	0.1 ± 0.3 0.0 0 to 1
ESSSDAI Mean ± SD Median Range		1.5 ± 1.3 1.0 0 to 5	2.6 ± 1.2 2.0 1 to 5	0.9 ± 0.9 1.0 ^a^ 0 to 3	2.0 ± 1.1 2.0 1 to 5	0.4 ± 0.9 0.0 0 to 3	2.8 ± 1.2 2.5 1 to 5	1.8 ± 1.0 1.5 1 to 3	1.5 ± 0.6 1.0 1 to 3	0.0 ± 0.0 0.0^d^ **0 to 0**
**Laboratory**										
ESR (mm Hg) Mean ± SD Median Range		21.3 ± 16.3 15.0 2 to 73	23.9 ± 15.7 22.0 2 to 59	19.5 ± 16.9 14.0 4 to 73	28.6 ± 18.0 26.0 6 to 73	12.1 ± 8.4 9.5^b^ 2 to 29	29.8 ± 14.6 27.0 8 to 59	7.0 ± 3.4 8.5^c^ 2 to 9	27.3 ± 21.8 16.0 6 to 73	13.8 ± 8.9 12.5 4 to 29
Serum IgM (mg/dL) Mean ± SD Median Range		208.1 ± 169.8 178.5 28 to 1170	186.1 ± 70.2 179.5 52 to 303	219.8 ± 204.3 178.5 28 to 1170	204.3 ± 90.1 186.0 70 to 553	214.6 ± 258.4 146.0 28 to 1170	198.3 ± 65.5 201.0 96 to 303	149.5 ± 80.9 148.0 52 to 250	208.5 ± 105.9 182.0 70 to 553	234.6 ± 292.5 137.0 28 to 1,170
Serum IgG (mg/dL) Mean ± SD Median Range		1779.8 ± 703.9 1,554.0 639 to 3,224	1973.4 ± 782.2 1,948.0 825 to 3,029	1676.6 ± 648.6 1,531.0 639 to 3,324	2092.1 ± 690.9 2,087.0 674 to 3,224	1247.1 ± 282.6 1,310.0^b^ 639 to 1,738	2261.7 ± 678.5 2,461.0 1,177 to 3,029	1108.5 ± 203.7 1,149.5^c^ 825 to 1,310	1972.4 ± 694.4 2,028.0 674 to 3,224	1289.8 ± 296.2 1,330.0^d^ 639 to 1,738
Serum IgA (mg/dL) Mean ± SD Median Range		345.7 ± 211.6 305.0 128 to 1420	394.1 ± 319.9 290.5 147 to 1420	319.9 ± 120.8 308.5 128 to 584	396.9 ± 248.9 343.0 128 to 1,420	258.5 ± 68.9 259.0 147 to 419	446.6 ± 355.8 337.0 176 to 1,420	236.8 ± 60.3 261.5 147 to 277	361.8 ± 135.0 353.0 128 to 584	265.2 ± 72.2 240.0 175 to 419
Serum hyperviscosity (AU) Mean ± SD Median Range		2.0 ± 0.5 1.9 1.6 to 4.3	2.2 ± 0.6 2.1 1.7 to 4.3	1.9 ± 0.3 1.8 1.6 to 2.5	2.2 ± 0.5 2.1 1.7 to 4.3	1.8 ± 0.1 1.8^b^ 1.6 to 2.0	2.3 ± 0.7 2.2 1.7 to 4.3	1.8 ± 0.2 1.7^c^ 1.7 to 2.0	2.0 ± 0.3* 1.9 1.7 to 2.5	1.8 ± 0.1 1.8^d^ 1.6 to 2.0
C3 (mg/dL) Mean ± SD Median Range		96.4 ± 23.6 92.6 53 to 147	92.1 ± 24.5 91.5 53 to 135	98.3 ± 23.3 92.9 60 to 147	88.5 ± 22.3 81.4 53 to 143	108.7 ± 19.9 105.8^b^ 77 to 147	88.7 ± 26.5 83.4 53 to 135	103.2 ± 14.2 99.0 92 to 119	88.4 ± 20.4 81.4 60 to 143	110.1 ± 21.3 106.4^d^ 77 to 147
C4 (mg/dL) Mean ± SD Median Range		19.8 ± 6.8 19.0 2 to 33	18.6 ± 9.6 19.1 2 to 33	20.3 ± 5.3 18.9 11 to 33	17.7 ± 6.8 18.6 2 to 33	23.6 ± 5.4 24.0^c^ 15 to 33	16.8 ± 10.2 18.5 2 to 33	24.4 ± 4.6 26.0 19 to 25	18.3 ± 4.0 18.6 11 to 51	23.4 ± 5.7 24.0 15 to 33
Leucocytes (cells/μL) Mean ± SD Median Range	6.4 ± 1.3 6.3 4.7--8.5	5.7 ± 2.0 5.5 1.5 to 12.6	5.6 ± 2.7 5.4 1.5 to 12.6	5.7 ± 1.7 5.7 3.0 to 10.7	5.1 ± 1.6 5.2 1.5 to 8.2	6.6 ± 2.5 6.2 3.9 to 12.6	4.8 ± 1.9 4.7 1.5 to 8.1	8.4 ± 3.4 7.8 5.2 to 12.6	5.4 ± 1.3 5.6 3.0 to 8.2	6.2 ± 2.0 6.0 3.9 to 10.7
Hemoglobin (g/dL) Mean ± SD Median Range	14.9 ± 0.8 14.9 13.3--16.5	13.9 ± 1.4 14.1 9.9 to 17.8	14.4 ± 1.3 14.0 12.7 to 17.8	13.7 ± 1.4 14.1 9.9 to 15.9	13.9 ± 1.1 14.0 12.1 to 15.9	14.1 ± 1.8 14.5 9.9 to 17.8	13.9 ± 0.7 14.0 12.7 to 15.0	16.1 ± 1.4 15.8 ^c^ 14.9 to 17.8	13.8 ± 1.3 14.1 12.1 to 15.9	13.6 ± 1.5 14.3 9.9 to 15.1
Platelets (cells/μL) Mean ± SD Median Range	264.2 ± 51.1 225.9 172--331	229.1 ± 68.1 229.5 73 to 367	233.7 ± 95.6 195.0 73 to 367	231.9 ± 50.0 231.0 149 to 357	223.9 ± 68.9 222.0 82 to 367	228.1 ± 69.3 221.0 73 to 357	240.2 ± 98.2 234.5 82 to 367	153.0 ± 56.8 172.0^c^ 73 to 195	212.4 ± 36.7 222.0 154 to 279	249.5 ± 57.5 245.5 149 to 357
Lymphocytes (%) Mean ± SD Median Range	31.1 ± 6.2 32.0 20.3--39.7	30.0 ± 10.8 28.0 9.0 to 58.0	29.9 ± 12.5 29.3 12.9 to 58	30.0 ± 9.9 27.5 9.0 to 52.3	31.1 ± 10.2 29.3 13.1 to 58.0	27.7 ± 12.3 26.5 9.0 to 52.3	33.5 ± 12.5 29.9 13.1 to 58.0	19.1 ± 7.3 18.2^c^ 12.9 to 27.0	29.5 ± 8.1 28.5 18.2 to 48.0	30.2 ± 12.5 27.1 9.0 to 52.3
Monocytes (%) Mean ± SD Median Range		8.0 ± 3.6 7.2 3 to 27	8.7 ± 5.2 7.1 5 to 27	7.6 ± 2.4 7.2 3 to 14	8.3 ± 4.2 7.8 5 to 27	7.4 ± 2.1 6.7 4 to 12	8.5 ± 6.0 7.1 5 to 27	7.9 ± 1.7 8.0 6 to 10	8.2 ± 2.4 8.0 5 to 14	7.2 ± 2.3 6.7 4 to 12
Polymorphonuclear cells(%) Mean ± SD Median Range		58.9 ± 11.8 61.9 15.0 to 79.5	59.5 ± 15.2 63.0 15.0 to 79.5	58.7 ± 9.8 61.7 38.8 to 76.0	57.3 ± 11.9 60.4 15.0 to 78.1	62.1 ± 12.0 64.4 38.8 to 79.5	56.0 ± 16.2 60.4 15.0--78.1	70.9 ± 6.9 70.6 63.0 to 79.5	58.3 ± 8.0 60.4 40.0 to 68.8	59.6 ± 12.2 63.5 38.8 to 76.0

Control, control group of healthy individuals. ^a^*P *< 0.05: patients with clinically active versus clinically inactive primary Sjögren's syndrome (pSS); ^b^*P *< 0.05: patients with serologically active versus serologically inactive pSS; ^c^*P *< 0.05: patients with clinically and serologically active versus clinically active but serologically inactive pSS; ^d^*P *< 0.05: patients with clinically inactive but serologically active versus clinically and serologically inactive pSS. SSDAI, Sjögren's syndrome disease activity index; ESSSDAI, European League Against Rheumatism (EULAR) Sjögren's syndrome disease activity index; ESR, erythrocyte sedimentation rate; Ig, immunoblobulin.

Forty-four percent (27/50) of patients had serological activity (with or without concomitant clinically active disease). There was no difference in the use of steroids or immunosuppressors in patients with and without serologic activity, no difference in the age of patients with active or inactive disease (49 versus 55 years, *P *= 0.07) and no difference in disease duration (10.9 versus 7.7 years, *P *= 0.24). However, as expected, patients with clinically active disease had higher SSDAI and ESSDAI scores (Table 1) and more frequently received prednisone (53% versus 6%, *P *= 0.001). On the other hand, patients with clinically active disease more frequently received immunosuppressors (58% versus 30%), but this difference was not statistically significant (*P *= 0.06). The frequency of medication use was as follows: azathioprine, 29% versus 3% (*P *= 0.01); methotrexate, 17% versus 6%; cyclophosphamide, 5% versus 3%, (*P *= 0.54); and antimalarial drugs, 35% versus 21% (*P *= 0.78), among patients with clinically active and inactive disease, respectively.

### Peripheral IL-10-producing B cells in patients with pSS

**Figure 2:**
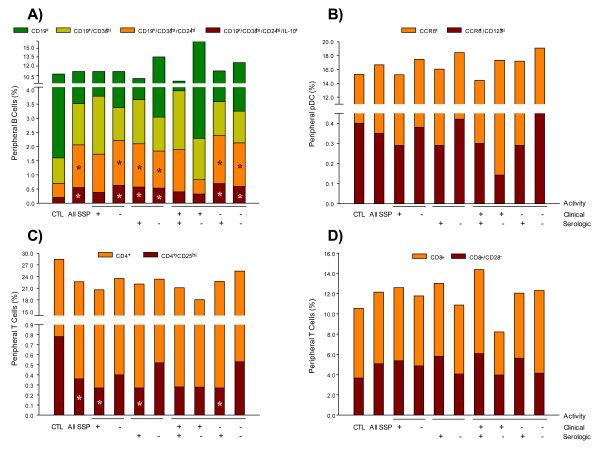
**Percentage of circulating cells**. (**A**) IL-10-producing B cells, (**B**) CCR6^+^/CD123^hi^-, (**C**) CD8^+^/CD28^-^- and (**D**) CD4^+^/CD25^hi^-circulating cells. Results are expressed as mean. *P < 0.05, for comparison of pSS patients with control (CTL) group.

To determine the different subtypes of IL-10-producing B cells, first CD19^+^/CD38^hi^/CD24^hi^/IL-10^+ ^immature/transitional circulating cells were identified and subsequently they were subphenotyped by determining the Ig expression on their surface (Table 2). Compared with healthy subjects, the CD19^+^/CD38^hi^/CD24^hi^/IL-10-producing B-cell subpopulation (0.20 ± 0.02% in healthy subjects) was greater in pSS patients with clinically inactive disease (0.63 ± 0.06%), with clinically active disease (0.55 ± 0.05%), with positive serology (0.57 ± 0.07%) and without serologic activity (0.53 ± 0.07%) (*P *< 0.05) (Table 2; Figure 2A). However, no differences were found in the frequency of circulating CD19^+^/CD38^hi^/IL-10^+ ^B cells among groups of patients (Table 2).

**Table 2:** IL-10-producing B cells, IDO-expressing dendritic cells and forkhead box protein 3-expressing regulatory T peripheral cells

Variable	Control (*n *= 25)	All pSS patients (*n *= 50)	Clinically active pSS patients (*n *= 17)	Clinically inactive pSS patients (*n *= 33)	Serologically active pSS patients (*n *= 29)	Serologically inactive pSS patients (*n *= 18)	Clinically and serologically active pSS patients (*n *= 12)	Clinically active and serologically inactive pSS patients (*n *= 4)	Clinically inactive and serologically active pSS patients (*n *= 17)	Clinically and serologically inactive pSS patients (*n *= 14)
CD19^+ ^B cells from PBMCs (%) Mean ± SEM Median Range	10.7 ± 0.7 10.5 5.2 to 19.3	11.0 ± 1.0 10.5 0.4 to 30.0	11.0 ± 1.6 10.7 1.6 to 27.3	11.0 ± 1.2 10.1 0.4 to 30.0	11.2 ± 1.3 9.4 0.4 to 30.0	13.0 ± 1.6 11.9 1.0 to 27.3	9.8 ± 2.1 10.0 1.6 to 27.3	15.6 ± 1.2 14.7 14.0 to 19.0	10.5 ± 1.7 8.4 0.4 to 30.0	12.2 ± 2.0 10.5 1.0 to 27.3
IL-10--producing B cells (%)										
CD19^+^/CD38^hi^ Mean ± SEM Median Range	1.6 ± 0.2 1.4 0.8 to 4.5	3.5 ± 0.3 2.9 0.3 to 9.0	3.8 ± 0.7 2.9 0.3 to 9.0	3.4 ± 0.3 2.9 1.2 to 8.8	3.8 ± 0.5 2.9 0.3 to 9.0	3.0 ± 0.3 2.8 1.2 to 5.8	4.0 ± 0.9 3.4 0.3 to 9.0	2.3 ± 0.5 2.1 1.3 to 3.6	3.6 ± 0.5 2.8 1.4 to 8.8	3.3 ± 0.3 3.0 1.2 to 5.8
CD19^+^/CD38^hi^/IL-10^+^ Mean ± SEM Median Range	0.13 ± 0.02 0.10 0.03 to 0.40	0.16 ± 0.02 0.12 0.03 to 0.91	0.18 ± 0.05 0.14 0.03 to 0.91	0.15 ± 0.02 0.11 0.03 to 0.43	0.17 ± 0.03 0.12 0.03 to 0.91	0.14 ± 0.03 0.08 0.03 to 0.35	0.21 ± 0.07 0.25 0.04 to 0.91	0.09 ± 0.05 0.07 0.03 to 0.21	0.14 ± 0.03 0.10 0.03 to 0.43	0.16 ± 0.03 0.13 0.03 to 0.35
CD19^+^/CD38^hi^/IgA^+^/IL-10^+^ Mean ± SEM Median Range	65.8 ± 1.5 66.4 51.3 to 78.6	75.7 ± 1.9 77.3^e^ 36.4 to 94.6	75.2 ± 3.6 74.6 45.8 to 94.6	76.3 ± 2.6 80.4^e^ 36.4 to 93.8	75.3 ± 2.6 78.1^e^ 36.4 to 94.6	75.3 ± 3.7 75.7^e^ 43.3 to 93.8	72.9 ± 4.5 74.4 45.8 to 94.6	77.7 ± 3.4 75.7 73.0 to 84.4	76.9 ± 3.2 80.0^e^ 36.4 to 89.8	74.5 ± 5.0 78.8 43.3 to 93.8
CD19^+^/CD38^hi^/IgG^+^/IL-10^+^ Mean ± SEM Median Range	55.3 ± 2.8 60.8 26.7 to 79.6	42.8 ± 2.0 41.7^e^ 22.2 to 74.2	38.6 ± 3.7 32.9^e^ 22.2 to 65.2	48.1 ± 2.0 48.6^ae^ 31.4 to 74.2	45.3 ± 2.5 41.7^e^ 23.2 to 74.2	45.2 ± 3.2 46.0 22.2 to 59.3	39.3 ± 4.2 32.9^e^ 23.2 to 65.2	41.2 ± 10.7 42.1 22.2 to 59.3	49.2 ± 2.9 49.5 31.4 to 74.2	46.6 ± 3.0 46.2 33.3 to 57.8
CD19^+^/CD38^hi^/IgM^+^/IL-10^+^ Mean ± SEM Median Range	21.7 ± 1.3 21.1 10.7 to 35.0	22.8 ± 1.5 20.2 3.0 to 54.0	20.2 ± 2.4 15.8 10.0 to 38.7	25.1 ± 2.2 22.7 3.0 to 54.0	22.8 ± 2.1 21.1 3.0 to 52.2	25.4 ± 3.2 23.1 12.1 to 54.0	21.7 ± 3.1 22.6 10.2 to 38.7	16.4 ± 1.7 15.8 13.8 to 19.5	23.5 ± 2.8 20.2 3.0 to 52.2	28.2 ± 3.8 28.2 12.1 to 54.0
CD19^+^/CD38^hi^/CD24^hi^ Mean ± SEM Median Range	0.7 ± 0.1 0.5 0.2 to 2.9	0.6 ± 0.1 0.5 0.1 to 1.6	1.7 ± 0.4 1.1 0.3 to 4.7	2.2 ± 0.3 2.0 0.4 to 7.7	2.2 ± 0.3 1.7 0.3 to 8.8	1.8 ± 0.2 1.7 0.4 to 3.7	1.9 ± 0.5 1.0 0.3 to 4.7	0.8 ± 0.2 0.8 0.4 to 1.3	2.4 ± 0.4 2.0 0.5 to 7.7	2.1 ± 0.3 2.2 0.5 to 3.7
CD19^+^/CD38^hi^/CD24^hi^/IL-10^+^ Mean ± SEM Median Range	0.20 ± 0.02 0.19 0.06 to 0.44	0.55 ± 0.05 0.46 ^e^ 0.12 to 1.59	0.38 ± 0.05 0.37 0.12 to 0.87	0.63 ± 0.06 0.56^e^ 0.13 to 1.59	0.57 ± 0.07* 0.47^e^ 0.12 to 1.59	0.53 ± 0.07 0.46^e^ 0.15 to 1.07	0.40 ± 0.06 0.40 0.12 to 0.87	0.32 ± 0.06 0.32 0.18 to 0.47	0.69 ± 0.09 0.65^e^ 0.13 to 1.59	0.59 ± 0.08 0.52^e^ 0.15 to 1.07
CD19^+^/CD38^hi^/CD24^hi^/CD5^+^/IL-10^+^ Mean ± SEM Median Range	17.7 ± 1.3 15.5 8.6 to 32.4	20.2 ± 1.4 20.0 2.8 to 45.2	11.4 ± 1.4 10.9^e^ 2.8 to 22.8	24.8 ± 1.4 23.7^a e^ 8.0 to 45.2	18.8 ± 1.7 18.8 2.8 to 38.7	22.9 ± 2.5 23.5 7.1 to 45.2	11.9 ± 1.8 11.8 ^e^ 2.8 to 22.8	11.0 ± 2.3 10.1 7.1 to 16.6	23.1 ± 2.0 22.0 8.0 to 38.7	27.1 ± 2.2 24.2^e^ 16.2 to 45.2
CD19^+^/CD38^hi^/CD24^hi^/CD10^+^/IL-10^+^ Mean ± SEM Median Range	15.8 ± 1.3 16.7 3.3 to 27.0	22.5 ± 1.6 22.9^e^ 4.4 to 42.9	16.9 ± 2.8 13.8 4.4 to 39.7	25.5 ± 1.7 25.6^ae^ 6.6 to 42.9	19.5 ± 2.0 20.0 4.4 to 39.7	27.5 ± 2.2 26.3^be^ 10.3 to 42.9	16.1 ± 3.9 10.7 4.4 to 39.7	19.6 ± 3.9 19.8 10.3 to 28.6	21.9 ± 2.1 22.9^e^ 6.6 to 33.3	30.1 ± 2.1 29.8^de^ 18.2 to 42.9
CD19^+^/CD38^hi^/CD24^hi^/CD20^+^/IL-10^+^ Mean ± SEM Median Range	20.3 ± 1.6 17.5 11.1 to 37.2	29.4 ± 2.1 27.9^e^ 10.2 to 66.7	23.8 ± 3.2 16.2 10.2 to 46.2	32.5 ± 2.6 30.9^ae^ 12.6 to 66.7	27.9 ± 2.5 23.8 12.5 to 54.8	28.5 ± 3.6 26.6 10.2 to 66.7	23.8 ± 3.5 16.5 12.5 to 45.5	18.4 ± 7.2 11.6 10.2 to 40.0	30.7 ± 3.3 33.0^e^ 12.6 to 54.8	31.2 ± 3.8 27.3^e^ 16.2 to 66.7
CD19^+^/CD38^hi^/CD24^hi^/CD27^+^/IL-10^+^ Mean ± SEM Median Range	35.4 ± 2.1 34.0 21.5 to 54.5	38.8 ± 1.8 41.0 11.3 to 68.5	37.0 ± 3.0 39.6 11.5 to 61.8	39.7 ± 2.3 41.0 11.3 to 68.5	40.1 ± 2.4 41.5 11.5 to 68.5	36.9 ± 2.7 37.9 11.3 to 52.5	37.5 ± 3.7 38.1 11.5--61.8	41.5 ± 1.8 43.0 37.9 to 43.6	42.0 ± 3.2 42.4 18.4 to 68.5	35.6 ± 3.2 35.0 11.3 to 52.5
CD19^+^/CD38^hi^/CD24^hi^/CD27^--^/IL-10^+^ Mean ± SEM Median Range	11.3 ± 1.8 7.8 0.0 to 32.6	23.9 ± 1.8 20.7^e^ 5.5 to 52.7	25.8 ± 3.8 22.4^e^ 5.5 to 52.7	23.0 ± 1.9 20.2^e^ 7.4 to 49.5	26.1 ± 2.5 21.7^e^ 5.5 to 52.7	20.5 ± 2.5 17.9^e^ 7.4 to 41.7	27.8 ± 4.9 28.2^e^ 5.5 to 52.7	21.5 ± 0.94 21.7 19.8 to 23.0	25.0 ± 2.6 20.7^e^ 9.8 to 49.5	20.7 ± 2.9 14.6^e^ 7.4 to 41.7
CD19^+^/CD38^hi^/CD24^hi^/CXCR4^+^/IL-10^+^ Mean ± SEM Median Range	28.3 ± 1.3 27.3 19.6 to 43.2	26.6 ± 1.5 27.0 9.6 to 46.1	23.0 ± 3.2 15.1 9.6 to 46.1	28.6 ± 1.4 28.9 16.0 to 44.4	25.4 ± 1.9 24.5 12.5 to 46.1	28.0 ± 2.6 28.1 9.6 to 44.4	21.8 ± 3.6 15.0 12.5 to 46.1	28.0 ± 9.1 29.0 9.6 to 44.4	28.1 ± 1.8 28.5 16.0 to 43.2	28.1 ± 2.1 28.5 17.2 to 44.4
CD19^+^/CD38^hi^/CD24^hi^/CXCR7^+^/IL-10^+^ Mean ± SEM Median Range	20.6 ± 1.3 18.7 12.3 to 33.1	25.9 ± 2.3 22.5 5.6 to 85.7	18.2 ± 2.5 17.3 5.6 to 34.62	29.5 ± 2.9 26.1^ae^ 7.3 to 85.7	24.3 ± 3.1 20.3 5.6 to 85.7	27.2 ± 3.0 27.2 10.8 to 50.0	17.4 ± 2.6 17.4 5.6 to 33.6	20.9 ± 7.1 17.2 10.8 to 34.6	28.8 ± 4.5 23.0 7.3 to 85.7	27.9 ± 3.2 27.2 17.1 to 50.0
pDC IDO^+ ^(%)										
CCR6^+^ Mean ± SEM Median Range	15.3 ± 0.9 16.5 8.7 to 21.8	16.7 ± 0.8 15.6 8.6 to 31.1	15.2 ± 1.1 15.4 8.6 to 24.7	17.5 ± 1.1 16.1 9.2 to 31.1	16.1 ± 1.0 15.5 8.6 to 28.7	18.4 ± 1.6 17.8 9.5 to 31.1	14.4 ± 1.3 14.3 8.6 to 24.6	17.3 ± 2.8 19.0 9.5 to 21.1	17.2 ± 1.3 16.1 9.2 to 28.7	19.1 ± 2.3 16.2 10.3 to 31.1
CD123^+^/CCR6^+^ Mean ± SEM Median Range	0.40 ± 0.06 0.35 0.12 to 0.98	0.35 ± 0.07 0.2 0.03 to 2.89	0.29 ± 0.06 0.2 0.07 to 0.82	0.38 ± 0.11 0.19 0.03 to 2.89	0.29 ± 0.34 0.2 0.03 to 1.84	0.42 ± 0.18 0.2 0.07 to 2.9	0.3 ± 0.06 0.21 0.08 to 0.72	0.14 ± 0.03 0.15 0.07 to 0.2	0.29 ± 0.10 0.15 0.03 to 1.84	0.46 ± 0.27 0.20 0.13 to 2.89
CD123^+^/CCR6^+^/IDO^+^ Mean ± SEM Median Range	16.6 ± 1.0 16.7 10.2 to 29.6	27.4 ± 1.8 26.7^e^ 7.4 to 57.2	30.8 ± 3.5 28.7^e^ 14.2 to 57.2	25.5 ± 2.0 23.9^e^ 7.4 to 49.4	28.0 ± 2.3 27.7^e^ 7.4 to 57.2	28.4 ± 3.2 28.2^e^ 12.1 to 49.4	31.4 ± 4.5 28.9^e^ 14.2 to 57.2	31.9 ± 6.0 32.8 ^c e^ 18.0 to 44.0	25.5 ± 2.4 27.4^e^ 7.4 to 43.0	27.2 ± 3.9 27.1^e^ 12.1 to 49.4
Foxp3--expressing T cells (%)										
CD4^+^ Mean ± SEM Median Range	28.4 ± 1.3 26.6 18.7 to 43.0	22.7 ± 1.4 22.7 7.1 to 45.7	20.6 ± 2.1 17.3 10.8 to 45.7	23.5 ± 1.8 25.2 7.1 to 39.8	22.1 ± 1.8 21.4 7.1 to 45.7	23.3 ± 2.5 26.1 8.6 to 39.8	21.1 ± 2.7 18.2 12.5 to 45.7	18.0 ± 4.1 15.7 10.8 to 29.9	22.8 ± 2.4 23.0 7.1 to 38.0	25.4 ± 3.3 26.9 8.6 to 39.8
CD4^+^/CD25^hi^ Mean ± SEM Median Range	0.78 ± 0.14 0.54 0.17 to 2.69	0.36 ± 0.06 0.26^e^ 0.10 to 2.93	0.27 ± 0.04 0.20^e^ 0.12 to 0.64	0.40 ± 0.09 0.27 0.10 to 2.93	0.27 ± 0.03 0.25^e^ 0.10 to 0.80	0.52 ± 0.17 0.28 0.12 to 2.93	0.28 ± 0.04 0.23 0.12 to 0.64	0.28 ± 0.09 0.28 0.12 to 0.44	0.27 ± 0.04 0.25^e^ 0.10 to 0.80	0.53 ± 0.24 0.28 0.12 to 2.93
CD4^+^/CD25^hi^/Foxp3^+^ Mean ± SEM Median Range	5.7 ± 0.3 5.8 3.3 to 9.0	6.4 ± 0.4 6.5 1.4 to 11.2	6.2 ± 0.6 5.9 2.2 to 11.2	6.5 ± 0.4 6.6 1.4 to 10.9	6.7 ± 0.5 6.9 2.0 to 11.6	6.1 ± 0.6 6.1 1.4 to 10.9	6.6 ± 0.8 6.4 2.2 to 11.2	6.2 ± 1.2 5.8 4.0 to 9.2	6.8 ± 0.6 6.9 2.0 to 10.4	6.0 ± 0.7 6.1 1.4 to 10.9
CD8^+^ Mean ± SEM Median Range	10.5 ± 0.8 10.6 3.5 to 16.7	12.2 ± 1.2 10.1 3.2 to 38.4	12.6 ± 2.5 8.7 3.2 to 38.4	11.8 ± 1.3 10.7 3.3 to 27.0	13.0 ± 1.8 9.7 3.2 to 38.4	10.9 ± 1.5 10.5 3.3 to 27.0	14.4 ± 3.5 10.1 3.2 to 38.4	8.2 ± 1.9 6.6 5.8 to 13.9	12.1 ± 2.0 9.7 3.5 to 25.6	12.3 ± 2.1 12.0 3.3 to 27.0
CD8^+^/CD28^--^ Mean ± SEM Median Range	3.7 ± 0.3 3.4 1.1 to 8.2	5.1 ± 0.6 3.6 0.8 to 17.4	5.4 ± 1.2 3.4 1.0 to 17.4	4.9 ± 0.6 3.7 0.8 to 13.6	5.8 ± 0.9 4.0 0.8 to 17.4	4.1 ± 0.6 3.6 1.4 to 9.3	6.1 ± 1.6 3.7 1.0 to 17.4	4.0 ± 1.3 3.5 1.4 to 7.4	5.6 ± 1.0 4.9 0.8 to 13.6	4.1 ± 0.7 3.6 1.6 to 9.3
CD8^+^/CD28^--^/Foxp3^+^ Mean ± SEM Median Range	5.6 ± 0.3 5.6 2.8 to 8.8	3.9 ± 0.3 3.7 ^e^ 1.1 to 8.9	4.2 ± 0.6 4.0 1.2 to 8.9	3.8 ± 0.3 3.6 ^e^ 1.1 to 7.3	3.9 ± 0.4 3.5 ^e^ 1.1 to 8.9	4.2 ± 0.5 4.4 1.8 to 8.5	4.0 ± 0.8 3.7 1.3 to 8.9	5.3 ± 1.2 5.1 2.6 to 8.5	3.8 ± 0.5 3.5 ^e^ 1.1 to 7.3	3.9 ± 0.5 3.9 ^e^ 1.8 to 6.8

Control, control group of healthy individuals. ^a^*P *< 0.05: patients with clinically active versus clinically inactive primary Sjögren's syndrome (pSS); ^b^*P *< 0.05: patients with serologically active versus serologically inactive pSS; ^c^*P *< 0.05: patients with clinically and serologically active versus clinically active but serologically inactive pSS; ^d^*P *< 0.05: patients with clinically inactive but serologically active versus clinically and serologically inactive pSS. IDO, indoleamine 2,3-dioxygenase; PBMCs, peripheral blood mononuclear cells; SEM, standard error of the mean; pDC, plasmacytoid dendritic cells.

**Figure 3:**
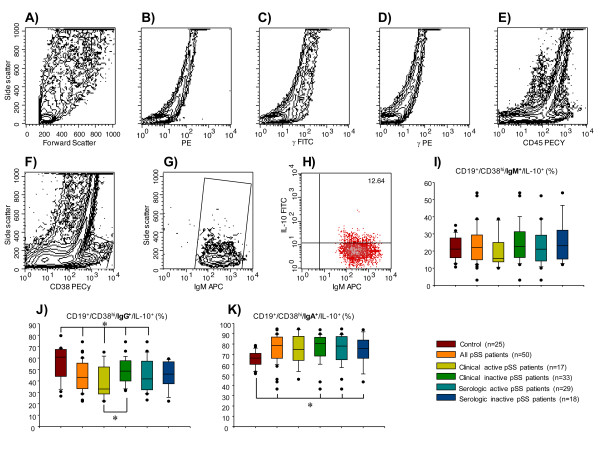
**Percentage of immunoglobulin (Ig) expression on IL-10-producing B peripheral-cell subtype in patients with primary Sjögren's syndrome (pSS)**. CD19^+ ^blood B cells were obtained by positive selection with microbeads. (**A**) Representative unstained and permeabilized sample of peripheral blood mononuclear cells (autofluorescence control). (**B**) phycoerythrin (PE)-labeled-anti rat-IL-10 IgG isotype control. (**C-E**) IgG_1_-fluorescein isothiocyanate (FITC)/IgG_1_-PE/CD45-PeCy5 mouse IgG_1_, k isotype controls (BD Tritest™, BD Biosciences). (**F**) Representative contour plot of CD19^+ ^B cells. An electronic gate was made for CD38^hi ^cells. (**G**) From the gate *F *CD19^+^⁄CD38^hi^/IgM^+ ^cells were determined. (**H**) From the latter CD19^+^⁄CD38^hi^⁄IgM^+^⁄IL-10^+ ^cells were defined. A total of 50,000 to 100,000 events were recorded for each sample before any gate setting, and were analyzed with the CellQuestPro software (BD Biosciences). Bar graphs show percentage of (**I**) CD19^+^⁄CD38^hi^⁄IgM^+^⁄IL-10^+ ^(**J**) CD19^+^⁄CD38^hi^⁄IgG^+^⁄IL-10^+^, and (**K**) CD19^+^⁄CD38^hi^⁄IgA^+^⁄IL-10^+ ^cells. (**I-K**) Results are expressed as median, 10^th^, 25^th^, 75^th^, and 90^th ^percentiles. *P < 0.05. APC, allophycocyanine.

For Ig surface expression, no changes were found in the CD19^+^/CD38^hi^/IgM^+^/IL-10^+ ^B-cell subpopulation between patient subgroups (Table 2; Figure 3I). However, the relative frequency of CD19^+^/CD38^hi^/IgG^+^/IL-10^+ ^was lower in all pSS subgroups when compared with the healthy control group (Table 2; Figure 3J). pSS patients with clinical activity had significantly lower levels of CD19^+^/CD38^hi^/IgG^+^/IL-10^+ ^compared to those with clinically inactive disease (38.6 ± 3.7 versus 48.1 ± 2.0%; *P *< 0.05) (Table 2; Figure 3J). With the exception of pSS patients with clinically active disease, all pSS subgroups had higher frequency of CD19^+^/CD38^hi^/IgA^+^/IL-10^+ ^B cells compared with the healthy control group (*P *< 0.05) (Table 2; Figure 3L**)**. It is important to recall that the results are expressed as the relative percentage obtained from CD19^+^/CD38^hi^/IL-10^+ ^B cells (Table 2).

**Figure 4:**
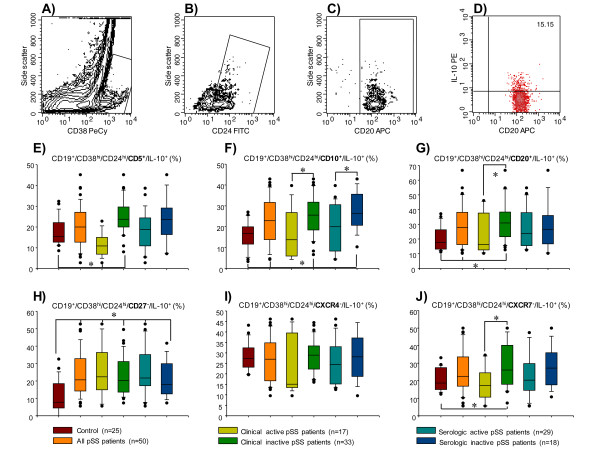
**Percentage of immature/transitional IL-10-producing B peripheral-cell subtype in patients with primary Sjögren's syndrome (pSS)**. CD19^+ ^blood B cells were obtained by positive selection with microbeads. (**A**) Representative contour plot of CD19^+ ^B cells. An electronic gate was made for CD38^hi ^cells. (**B) **From the gate *A *CD19^+^⁄CD38^hi^⁄CD24^hi ^cells were determined. (**C**) From the gate *B *CD19^+^⁄CD38^hi^⁄CD24^hi^⁄CD20^+ ^were defined. (**D**) From the latter CD19^+^⁄CD38^hi^⁄CD24^hi^⁄CD20^+^⁄IL-10^+ ^cells were determined. Bar graphs show percentage of (**E**) CD19^+^⁄CD38^hi^⁄CD24^hi^⁄CD5^+^⁄IL-10^+^, (**F**) CD19^+^⁄CD38^hi^⁄CD24^hi^⁄CD10^+^⁄IL-10^+^, (**G**) CD19^+^⁄CD38^hi^⁄CD24^hi^⁄CD20^+^⁄IL-10^+^, (**H**) CD19^+^⁄CD38^hi^⁄CD24^hi^⁄CD27^-^⁄IL-10^+^, (**I) **CD19^+^⁄CD38^hi^⁄CD24^hi^⁄CXCR4^+^⁄IL-10^+^-, and (**J**) CD19^+^⁄CD38^hi^⁄CD24^hi^⁄CXCR7^+^⁄IL-10^+^-producing B peripheral cells. A total of 50,000 to 100,000 events were recorded for each sample before any gate setting and were analyzed with the CellQuestPro software (BD Biosciences). (**E-J**) Results are expressed as median, 10^th^, 25^th^, 75^th^, and 90^th ^percentiles. *P < 0.05.

The frequency of immature/transitional CD19^+^/CD38^hi^/CD24^hi^/CD5^+^/IL-10^+ ^B cells was higher in patients without clinical activity and lower in patients with clinical activity when compared with the control group (*P *< 0.05) (Table 2; Figure 4E). The percentage of CD19^+^/CD38^hi^/CD24^hi^/CD10^+^/IL-10^+ ^was higher in pSS patients who had both clinical and serologic inactivity when compared to healthy subjects (*P *< 0.05) (Table 2; Figure 4F). Moreover, CD19^+^/CD38^hi^/CD24^hi^/CD10^+^/IL-10^+ ^circulating B cells were higher in pSS patients with clinically inactive versus clinically active disease, and in pSS patients without serologic activity versus pSS patients with serologic activity (*P *< 0.05) (Table 2; Figure 4F). The percentage of CD19^+^/CD38^hi^/CD24^hi^/CD20^+^/IL-10^+ ^was higher in pSS patients with clinically inactive disease when compared to healthy subjects, and to pSS patients with clinically active disease (*P *< 0.05) (Table 2; Figure 4G).

In addition, a different IL-10 B subset, a non-memory B cell that could be the human counterpart of mouse marginal-zone B cells, a CD19^+^/CD38^hi^/CD24^hi^/CD27^+^B10 subset was also immunophenotyped. No statistically significant differences were found in the B10 subset between pSS patients and the control group (Table 2). Nonetheless, the percentage of CD19^+^/CD38^hi^/CD24^hi^/CD27^-^/IL-10^+ ^cells was increased in pSS patients independently of clinical or serologic activity (*P *< 0.050 (Table 2; Figure 4H). The percentage of CD19^+^/CD38^hi^/CD24^hi^/CXCR4^+^/IL-10^+ ^cells was similar in pSS patients and the healthy control group (Table 2; Figure 4I). The cell frequency of CD19^+^/CD38^hi^/CD24^hi^/CXCR7^+^/IL-10^+ ^was higher in pSS patients without clinical activity when compared with pSS patients with clinically active disease and healthy controls (*P *< 0.05) (Table 2; Figure 4J). It is important to note that the results are expressed as the relative percentage obtained from CD19^+^/CD38^hi^/CD24^hi^/IL-10^+ ^B cells (Figure 2A, red bars).

### Percentage of IDO^+ ^plasmacytoid dendritic cells in patients with pSS

**Figure 5:**
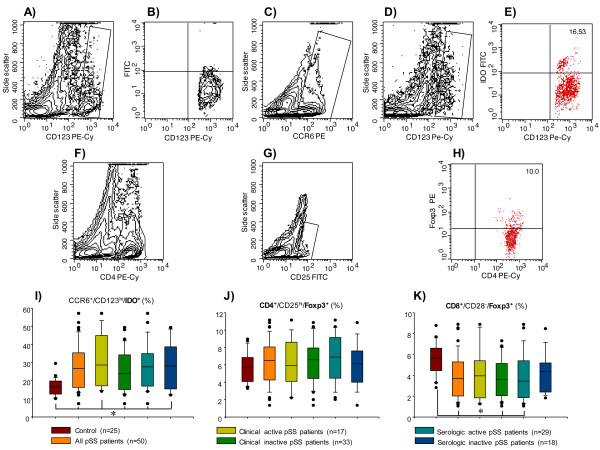
**Percentage of indoleamine 2,3-dioxygenase (IDO)- and Foxp3-expressing peripheral blood cells in patients with primary Sjögren's syndrome (pSS)**. (**A,B**) Control of fluorescein isothiocyanate (FITC)-labeled-rabbit anti-sheep specificity staining. **(C) **An electronic gate was made for CCR6^+ ^cells. (**D**) From the gate *C *CCR6^+^⁄CD123^hi ^cells were determined. (**E**) From the latter CCR6^+^⁄CD123^hi^⁄IDO^+ ^cells were defined. (**F**) An electronic gate was made for CD4^+ ^cells. (**G**) From the gate *F *CD4^+^⁄CD25^hi ^were determined and an electronic gate was made for double positive cells. (**H**) From the latter CD4^+^⁄CD25^hi^⁄Foxp3^+ ^cells were defined. Bar graphs show percentage of (**I**) CCR6^+^⁄CD123^hi^⁄IDO^+ ^cells, (**J**) CD4^+^⁄CD25^hi^⁄Foxp3^+ ^cells, (**K**) CD8^+^⁄CD28^-^⁄Foxp3^+ ^cells. A total of 100,000 to 250,000 events were recorded for each sample before any gate setting, and were analyzed with the CellQuestPro software (BD Biosciences). (**I-K**) Results are expressed as median, 10^th^, 25^th^, 75^th^, and 90^th ^percentiles. *P < 0.05.

A subpopulation of plasmacytoid DCs derived from monocytes expressing CD123^hi^/CCR6^+^/IDO^+ ^that are responsible for mediating the suppression of effector T cells has recently been described. The frequency of these cells in peripheral blood was higher in all subgroups of patients with pSS when compared with the control group (*P *< 0.05) (Table 2**; **Figure 5I).

### Frequency of Foxp3^+ ^regulatory T cells circulating in patients with pSS

We performed analyses to determine the various subpopulations of lymphocytes that regulate the adaptive immune response and induce peripheral tolerance of CD4^+^/CD25^hi^/Foxp3^+ ^and CD8^+^/CD28^-^/Foxp3^+ ^cell subpopulations. There were no statistically significant differences in the frequency of CD4^+^/CD25^hi^/Foxp3^+ ^cells (Table 2; Figure 5J). Nevertheless, a lower percentage of CD8^+^/CD28^-^/Foxp3^+ ^cells was observed in pSS patients without clinically active disease but with serologic activity compared with the healthy control group (*P *< 0.05) (Table 2; Figure 5K).

## Discussion

For a long time, pSS was considered a Th1 autoimmune disease, and this lymphocyte subset has been the protagonist of immune damage. Nevertheless, the contemporary view of pSS considers the B cell population as preponderant in its pathogenesis [22]. Nevertheless, this new hypothesis does not consider regulatory B cells, probably due the recent description or the scarce information about their role in pSS. Recently, however, phenotypic and functional features of these novel IL-10-producing B cells have been described, for example, they are potent suppressors of Th1 cell differentiation (through cell-cell contact and production of IL-10), are inhibitors of autoimmune pathogenesis, and importantly, can restore immune system homeostasis [31]. It is worth mentioning that regulatory B cells represent only 0.2 to 0.6% of total B cells, suggesting that its regulatory potential is highly effective.

The objective of this study was to characterize three subpopulations of regulatory cells namely, IL-10-producing B cells, Foxp3-expressing T cells and IDO-expressing DCs in 50 patients with clinically active or inactive and serologically active or inactive pSS. Unexpectedly, CD4^+^/CD25^hi^/Foxp3^+ ^regulatory T cells were comparable between all pSS groups, suggesting that, at least systemically, these cells are not affected by clinical or serologic pSS activity and, moreover, do not appear to be an important peripheral cell biomarker. Furthermore, in an indirect way, these data support the hypothesis of the key role B cells play in disease pathogenesis [22,31,32].

Furthermore, a previous study reported that the number of infiltrating CD4^+^/CD25^hi^/Foxp3^+ ^cells positively correlated with the minor salivary gland biopsy focus score and negatively correlated with the prevalence of these cells in the peripheral blood, suggesting a reverse regulation in the periphery and the affected tissue. Nevertheless, in patients with advanced histologic lesions, high percentages of Treg cells were detected in peripheral blood [33]. According to our data, we also support the low prevalence of these circulating cells suggesting a reverse peripheral regulation and injured tissue. On the other hand, our work provides evidence for the presence of another subpopulation of naturally occurring regulatory T cells, the CD8^+^/CD28^-^/Foxp3^+ ^cell subset. They were found at lower levels in all pSS patient groups when compared with the healthy control group, suggesting that IL-10-producing regulatory CD8^+ ^T cells might be migrating to the lacrimal and salivary glands, as a result of their regulatory effect, which appeared to be mediated by direct contact with target cells [32]. Foxp3-expressing CD8^+^/CD28^- ^Tregs share developmental and phenotypic features (CD122^+^/GITR^+^/CTLA4^+^/CCR7^+^/CD62L^+^/CD25^+^/CD127^-^/IL-23R^-^) with naturally occurring CD4^+ ^Tregs. Secretion of IL-10 and transforming growth factor (TGF)-β1 is higher in CD8^+^/CD25^hi^/CD28^-^/Foxp3 Tregs than in CD8^+^/CD25^hi^/CD28^- ^T cells. In addition, Foxp3-expressing CD8^+ ^Tregs reduce T cell proliferation in response to a specific antigen and secretion of both IFN-γ and IL-17 by CD4 T cells only through cell-cell contact. On the other hand, CD8^+ ^Tregs downregulate the co-stimulatory molecule expression on DCs (CD40, CD80, CD86, MHC I, HLA-DR), inducing a less efficient antigen presentation [32,34,35]. It has previously been demonstrated that CD8^+^/CD28^-^/Foxp3^+ ^regulatory T cells can condition DCs to express functional and suppressive IDO activity [32]. In turn, IDO-expressing pDCs have the capacity to suppress T-cell responses to allo- and auto-antigens [36]. In this study, an increase in the percentage of CD123^hi^/CCR6^+^/IDO^+ ^pDC was observed in all pSS patient groups when compared with the peripheral frequency observed in healthy subjects. The role of IDO and the specific IDO-expressing cells in normal and disease conditions has not yet been fully characterized. CD123^hi^/CCR6^+^/IDO^+ ^pDC constitute only 0.2 to 0.8% of peripheral blood cells and represent a unique, rather plastic, versatile, and important immune cell population capable of producing over 95% of IFN-I synthesized by PBMC in response to virus and nucleic acid-containing complexes from the host [37]. In fact, IFN-I upregulation has been shown in both peripheral cells and salivary glands from pSS patients, the so-called IFN signature [38], and it has been postulated that a primary viral infection induces IFN-I synthesis in salivary glands with subsequent activation of the adaptive immune response resulting in autoantibody production against RNA binding proteins, for example, SSA, SSB, RNP, which are interferogenic complexes that stimulate CD123^hi^/CCR6^+^/IDO^+ ^cells to synthesize IFN-I, although this does not distinguish clinical disease activity. Thus, DCs could play a dual role in pSS pathogenesis: regulation of the immune response and IFN-I production.

In addition to several Tregs and DCregs, our results show that human peripheral blood has at least one more tolerogenic subset, namely IL-10-secreting B cells. Mauri and colleagues have defined these latter cells as a regulatory B-cell pool with many subtypes that display a CD19^+^/CD24^hi^/CD38^hi^/IL-10^+ ^phenotype [31]. Iwatta *et al. *previously reported that CD19^+^/CD24^hi^/CD38^hi^/CD27^+^/IL-10^+ ^B10-cell frequencies in blood from a group of patients with autoimmune diseases, such as lupus, rheumatoid arthritis, SS, autoimmune skin disease, and multiple sclerosis, were not significantly different from those observed in healthy controls, although mean B10 and B10 pro-cell frequencies were significantly increased. However no further characterization for each disease was provided [39].

Herein, the regulatory B-cell subset percentage showed clear differences in pSS patients with active versus inactive disease. CD19^+^/CD24^hi^/CD38^hi^/IL-10^+ ^cells were significantly higher in the whole pSS group compared to HD. However, this difference was at the expense of pSS patients with clinically inactive disease, where IgA-expressing regulatory B cells have higher peripheral frequency. IgA secretion by the salivary gland in pSS patients suggests that its peripheral blood occurrence is a clear systemic reflection of what might be happening at the site of injury. Our suggestion is based on work by Cerutti's group who have clearly shown that functional toll-like receptor (TLR)3 is expressed by human tonsillar B-cells, with higher expression in GCs and sub-epithelial regions, but is absent from memory B cells (CD27^+ ^cells). In the presence of dsRNA, these mucosal B cells upregulate AID expression and initiate class switch recombination and IgG/IgA production in the presence of IL-10 and B cell activating factor (BAFF) [40]. Surprisingly, IgG-expressing regulatory B cells had lower peripheral frequency in patients compared to healthy individuals. The lowest frequency was found in pSS patients with clinically active versus inactive disease. There was no difference between patients and controls in surface IgM expression.

Recently, a CD19^+^/CD24^hi^/CD38^hi^/CD5^hi ^B-cell subtype has been described. CD5 itself has been demonstrated to affect B-cell function by negative regulation of B-cell receptor (BCR) signaling, as well as by inducing the production of IL-10 [41]. CD5^+ ^IL-10-producing B cells suppress the proliferation of Th1 through CD40 engagement, and STAT3 phosphorylation. Meanwhile, the differentiation of Th1 cells is inhibited in an IL-10-dependent, but TGF-β1-independent manner, which requires CD80/CD86 interactions with target CD4^+ ^T-cells. In addition to halting Th1 but not Th17 responses, the suppressive effects are mediated by an indirect mechanism, through the induction of Foxp3^+ ^expression in CD4^+^/CD25^hi ^T cells [4,9] in a more efficient way than any other population of antigen-presenting cells [5,6]. The resulting Tregs displayed a greater suppressive capacity than regulatory T-cells generated by immature DCs from the same donor [7]. This suggests that B cell-dependent suppressive effects are associated with the generation of Foxp3-expressing CD4^+^/CD25^hi ^Tregs. In our study we found higher proportions of CD19^+^/CD24^hi^/CD38^hi^/CD5^+ ^IL-10-secreting B cells in pSS patients with clinically inactive disease compared to those with clinically active disease, the whole pSS group, and healthy controls, suggesting that CD5^+ ^IL-10-producing B cells are able to downregulate autoimmune inflammation to induce homeostasis [5-8].

Another B cell subpopulation in our patients was that of CD19^+^/CD38^hi^/CD24^hi^/CD10^+^/IL-10^+ ^cells. CD10 is a cell membrane metallopeptidase expressed by early B, pro-B, and pre-B lymphocytes and diffuse large B cells. CD10 expression is a well-accepted marker for most of the transitional T1/T2 B-cell pool, suggesting that these cells are recent emigrants from the bone marrow [4]. Our findings show a statistically significant increase in the frequency of these B cells in pSS patients with clinically inactive, serologically inactive, and clinically and serologically inactive disease compared to pSS patients with clinically active, serologically active, and clinically and serologically active disease, and healthy controls, attributable to a more immature differentiation stage of these cells (probably T2) [42].

The levels of the CD19^+^/CD38^hi^/CD24^hi^/CD20^+^/IL-10^+ ^B-cell subset were higher in the whole pSS group, and in patients with clinically inactive, and clinically and serologically inactive disease compared to healthy controls. Moreover, levels of CD20^+^/IL-10^+ ^B cells were significantly lower in pSS patients with clinically active versus clinically inactive disease. CD20 is a 33-kd phosphoprotein similar to an ion channel that allows calcium influx for cell activation. It is expressed on pre-B and mature B cells after CD19/CD10 expression, and before CD21/CD22 and surface Ig expression. It is retained on mature B cells until plasma cell development (plasmablasts) [5]. It has a central role in the generation of T cell-independent (TI) antibody responses. Although antigen-independent B cells have been shown to develop normally, in the absence of CD20 expression, antibody formation, particularly after vaccination with TI antigens, is strongly impaired in deficient patients [43]. In this sense, we suggest that the decrease in CD20 expression in patients with active pSS could be an immune regulatory mechanism that is able to avoid autoimmune antibody response and maintain the IL-10-producing B cell tolerant phenotype.

In addition to the human CD19^+^/CD24^hi^/CD38^hi^-circulating B-cell subpopulation aforementioned, it has been suggested that CD19^+^/CD38^hi^/CD24^hi^/CD27^+^/IL-10^+ ^or B10 cells might be a different Breg subset, as described by Tedder and colleagues [39]. They are present in the splenic marginal zone rather than memory cells generated in germinal centers. Whereas CD40/CpG-stimulated B10 cells induce proliferation and produce higher levels of IL-10 (10-fold) compared to CD27^- ^(cells that had not yet entered the germinal center), only B10 cells inhibit mitogen-induced TNF-α production by monocytes, through IL-10 synthesis [39,44-48], suggesting neither a non-inflammatory nor a viral infectious process.

Remarkably, when compared to healthy controls, there were higher levels of naïve and transitional CD19^+^/CD38^hi^/CD24^hi^/CD27^-^/IL-10^+ ^B cells in all pSS patient groups except for the group with clinically active and serologically inactive disease. We consider that the relative increase in CD27^- ^peripheral B cells could be originated by a B-cell exhaustion mechanism, where accumulation of peripheral blood CD27^- ^tissue-like B cells is a consequence of persistent chronic immune activation (autoimmune disease) or persistent viral infections. The features of CD27^- ^B cells include increased expression of multiple inhibitory receptors, decreased cell function, and poor proliferative and effector responses to a variety of stimuli [49].

Among all chemokine receptors, CXCR4 induces prolonged activation of intracellular signal transduction pathways, such as the mitogen-activated protein kinase (MAPK) cascade. This may elicit anti-apoptotic responses and thus, contribute to cell survival. In B-cell lymphopoiesis, CXCR4/CXCL12 are critical for bone marrow retention and maturation of the cells [50]. In the present study, there were no differences between CD19^+^/CD38^hi^/CD24^hi^/CXCR4^+^/IL-10^+ ^levels in pSS patients and controls, suggesting that, at least at systemic level, these cells are not affected by clinical or serologic pSS activity: CD19^+^/CD38^hi^/CD24^hi^/CXCR7^+^/IL-10^+ ^regulatory B cells were increased in pSS patients with clinically inactive disease compared to patients with clinically active disease and healthy controls. CXCR7 is a receptor for chemokines CXCL12/SDF1 and CXCL11. It does not elicit classical chemokine receptor signaling; chemokine binding does not activate G-protein-mediated signal transduction, but instead induces beta-arrestin recruitment, leading to ligand internalization and activation of the MAPK signaling pathway. The CXCR7 receptor acts as a scavenger for CXCL12/SDF1 and, to a lesser extent, for CXCL11. It is required for regulation of CXCR4 protein levels in migrating interneurons, thereby adapting their chemokine responsiveness. CXCR7 promotes cell growth and survival. It is not involved in cell migration, adhesion or proliferation of normal hematopoietic progenitors [51,52]. Thus, we suggest that CXCR7 promotes survival of IL-10-producing B cells, and CXCR4 could participate in migrating cells to the site of inflammation, and may perhaps interact *in situ *with pro-inflammatory cells.

Clinical disease activity necessarily implies the presence of an exacerbation trigger (still unknown) that might overcome the regulatory B effect by the increase of pathogenic B cells with a concomitant decrease of regulatory B cells. This certainly leads to an uncontrolled autoimmune process and loss of homeostasis. B-cell modulation is clearly a promising therapy in SS. For instance, rituximab (anti-CD20 monoclonal antibody) has been employed for the treatment of extraglandular manifestations, features of SS, and fatigue in pSS with controversial results in controlled clinical trials [53]. It eliminates almost all peripheral B cells, again supporting the notion that B cells are the preponderant cells at play in pSS pathogenesis. In the same vein, BAFF is a cytokine clearly implicated in the pathogenesis of SS. Recently, results of a first open, phase 2 study of belimumab, a monoclonal anti-BAFF antibody, showed an improvement in clinical activity assessed by the ESSDAI and ESSPRI indexes [54]. Caution should be taken, because in theory both drugs may also affect the Breg cells and could exacerbate inflammation and autoimmunity.

There are potential limitations to the current study. First, as expected, the group with clinically active disease received steroids and immunosuppressors more frequently (although overall, the latter were not statistically significant when compared to the group with clinically inactive disease); whether or not the impact of the treatment influences the function of regulatory cells is matter of debate. However, we consider that our results are still valid and robust because in the same vein, rheumatoid arthritis patients with inactive disease have also been recently reported as having an increased number of CD19^+^CD24^hi^CD38^hi ^B cells compared with patients with active disease [55]. Second, this work was a cross-sectional study. Thus, longitudinal studies may be necessary for a better understanding of causality.

Nevertheless, as far as we know, this is the first study that evaluated the presence of three regulatory cell subpopulations, particularly, IL-10-producing B cells in pSS patients. Undoubtedly, the immunobiology of regulatory B cells is still young and much remains to be learned about regulatory mechanisms. Next, evaluation should consider exploring ontogeny and population diversity, differentiation pathways, transcription factor(s), specific surface markers, plasticity and functionality of the cells involved. Our preliminary results deserve to be studied in depth to appraise the clinical relevance of these findings. In this vein, the evaluation of these cells in salivary glands is ongoing, to confirm peripheral blood findings, and certainly functional studies will be needed.

## Conclusion

In general it can be concluded that the levels of regulatory B-cell phenotypes studied were greater in patients with clinically inactive pSS and serologically inactive pSS. These findings emphasize two important Breg functions: the control of autoimmune disease and restoration of immune homeostasis. Moreover, Breg immunophenotyping appears to be a reliable marker of cell stability and control of the disease, which may be useful for monitoring those patients who progress to lymphoma.

## Abbreviations

APCs: antigen-presenting cells; APC: allophycocyanine; BAFF: B cell activating factor; BCR: B cell receptor; CpG: cytosine-phopsphate-guanosine; DC: dendritic cell; DCregs: indoleamine 2,3-dioxygenase-expressing dendritic cells; ESSDAI: European League Against Rheumatism Sjögren's syndrome disease activity index; ESR: erythrocyte sedimentation rate; EULAR: European League Against Rheumatism; FACS: fluorescence-activated cell sorting; FITC: fluorescein isothiocyanate; GITR: glucocorticoid-induced tumor necrosis factor receptor; HD: healthy donors; HLA-DR: human leukocyte antigen-DR; IDO: indoleamine 2,3-dioxygenase; IFN-γ: interferon-γ; Ig: immunoglobulin; IL: interleukin; iNKT: inducible natural killer T cells; MAPK: mitogen-activated protein kinase; MHC I: major histocompatibility complex I; NK: natural killer; PBMC: peripheral blood mononuclear cells; PE: phycoerythrin; PE Cy5: phycoerythrin and Cyanine 5; pDCs: plasmacytoid dendritic cells; pSS: primary Sjögren's syndrome; RNP: ribonucleoprotein; SEM: standard error of the mean; SLE: systemic lupus erythematosus; SSA: Ro/SSA antigen; SSB: La/SSB antigen; SSDAI: Sjögren's syndrome disease activity index; STAT3: signal transducer and activator of transcription 3; TGF-β1: transforming growth factor β1; Th: T helper; TLR: toll-like receptor; TNF-α: tumor necrosis factor-α; Tr1: IL-10-producing Treg cells; Tregs: regulatory Foxp3-expressing T cells.

## Competing interests

The authors declare that they have no competing interests.

## Authors' contributions

JFC, GL, GHM, and LL conceived and designed the experiments. JFC, GL, YRV, and KFB performed the experiments. JFC, GL, and GHM analyzed the data. JFC, GHM, GL, YRV, and LL contributed reagents/materials/analysis tools. JFC, GL, GHM, and LL wrote the paper. All authors read and approved the final manuscript.
